# Melanoma genomics – will we go beyond *BRAF* in clinics?

**DOI:** 10.1007/s00432-024-05957-2

**Published:** 2024-09-28

**Authors:** Justyna Mirek, Wiesław Bal, Magdalena Olbryt

**Affiliations:** 1 Center for Translational Research and Molecular Biology of Cancer, Maria Sklodowska-Curie National Research Institute of Oncology Gliwice Branch, Gliwice, 44-101 Poland; 2 Chemotherapy Day Unit, Maria Sklodowska-Curie National Research Institute of Oncology Gliwice Branch, Gliwice, 44-101 Poland

**Keywords:** Melanoma, Genomics, NGS, Genomic testing, BRAF

## Abstract

In the era of next-generation sequencing, the genetic background of cancer, including melanoma, appears to be thoroughly established. However, evaluating the oncogene *BRAF* mutation in codon V600 is still the only companion diagnostic genomic test commonly implemented in clinics for molecularly targeted treatment of advanced melanoma. Are we wasting the collected genomic data? Will we implement our current genomic knowledge of melanoma in clinics soon? This question is rather urgent because new therapeutic targets and biomarkers are needed to implement more personalized, patient-tailored therapy in clinics. Here, we provide an update on the molecular background of melanoma, including a description of four already established molecular subtypes: BRAF+, NRAS+, NF1+, and triple WT, as well as relatively new NGS-derived melanoma genes such as *PREX2*, *ERBB4*, *PPP6C*, *FBXW7*, *PIK3CA*, and *IDH1*. We also present a comparison of genomic profiles obtained in recent years with a focus on the most common melanoma genes. Finally, we propose our melanoma gene panel consisting of 22 genes that, in our opinion, are “must-have” genes in both melanoma-specific genomic tests and pan-cancer tests established to improve the treatment of melanoma further.

## Introduction

Melanoma is the most aggressive skin cancer characterized by high metastasis risk and poor prognosis in the advanced stage. Only 30–35% of metastatic patients have a chance of surviving five years when treated with modern drugs (Buzaid and Gershenwald 2023). The main risk factors for melanoma development are sun exposure, abnormal nevi, skin pigmentation phenotype, age, and a family history of malignancy. The main hereditary factors contributing to melanoma development are mutations in the *CDKN2A* or *CDK4* gene, polymorphism of *MC1R*, and many nevi. Hereditary melanomas constitute 5–12% of all melanomas, while UV radiation accounts for most of melanoma cases (Davis et al. 2019).

Melanoma is curable in its early stage. However, when disseminated to distant organs, in most cases, it is a fatal disease. Nowadays, there are two treatment modalities for advanced melanoma: molecular targeted therapy (inhibitors of BRAF and MEK kinases; BRAFi/MEKi) and immunotherapy with the usage of immune checkpoint inhibitors (anti-PD-1, -CTLA-4, and -LAG-3 antibodies). Implementing these therapeutic strategies has revolutionized melanoma treatment, extending median survival time from 7 months to approximately 25–72 months in advanced stage (Robert et al. 2019; Wolchok et al. 2022). However, many patients still do not benefit from the treatment, and the vast majority of them will eventually experience a relapse or progression within the 5-year follow-up. Thus, the main challenges in advanced melanoma management are the identification of novel therapeutic targets and predictive biomarkers for standard therapies and the development of personalized, patient-tailored treatment. Better knowledge of melanoma biology, including its genetic background, has already contributed significantly to progress in melanoma treatment; however, still much knowledge is to be discovered and implemented in the clinic. Here, we present up-to-date data on melanoma genomics with a particular focus on its present and potential usage in melanoma treatment. Based on the current knowledge, we try to answer the question of whether other melanoma genomic tests can enrich current *BRAF* diagnostics in the clinics.

## The genomic landscape of melanoma

Malignant melanoma is characterized by high heterogeneity and the highest number of somatic mutations (APGI et al. 2013) with approximately 14.0–17.0 mutations/Mb in coding DNA (Hodis et al. 2012; TCGA Network 2015) and 72 mutations per Mb in the whole genome (Newell et al. 2022). The main environmental factor inducing mutations in melanocytes is ultraviolet radiation. UV mutation signature is characterized by a higher prevalence of C > T substitutions at dipyrimidine sites in melanoma DNA, especially those localized in the skin (Brash 2015). Exposure to UV radiation, as well as localization of melanoma, determine its genetic background, which is specific to four melanoma subtypes: uveal (UM), acral (AM), mucosal (MM), and cutaneous (CM) (Hayward et al. 2017; Newell et al. 2022). These four histological types of melanoma differ in the number of mutations, with uveal being the least mutated and cutaneous the most, as well as with mutational signature, type of variants (rearrangements, amplifications, and SNVs), and genomic profile. COSMIC SBS signatures caused by UV exposure (SBS7a-d) prevail in CM, while non-CM types are dominated by age-associated signatures: SBS1, SBS5, and SBS40. UM is the most genetically homogenous melanoma with few mutations (mean 0.5/Mb) and rearrangements (mean 9). The latter ones are mainly deletions in the chromosome 3 or amplifications in the chromosome 8. On the contrary, other melanoma types are characterized by lots of complex structural rearrangements and chromosomal instability, with AM and MM having the most amplifications and deletions. In their complex work, Newell et al. (2022) identified many other genetic associations e.g., *NF1*-mutated CM localized in the head and neck has the highest tumour mutation burden (TMB), AM and MM have a lower ratio of TMB but more amplifications and breakpoints in the genome in comparison to CM; *TERT* promoter mutations were more common in CM (84%) than in AM (10%) and MM (16%). The authors also analyzed specific genomic profiles within those four melanoma types using previously identified The Cancer Genome Atlas (TCGA) molecular categories: BRAF+, RAS+, NF1+, and triple negative. Again, UM proved to have a unique, unlike other melanomas, genomic profile. The genomic and histopathological subtypes and the differences in UV mutational signature have been thoroughly described by Rabbie et al. (2019). Here, we would like to focus on the most common type – cutaneous melanoma (CM) and present up-to-date knowledge of CM genomics and its current and potential clinical implications.

Since the advent of next-generation sequencing, our knowledge of the genomic background of melanoma has increased significantly. This high throughput and sensitive technique enabled the identification of many low-frequency variants in both, the population and the tumours, that were undetectable so far (Hodis et al. 2012; Zhang et al. 2016). The landmark genomic study is the next-generation sequencing (NGS) analysis of cutaneous melanoma samples derived from 331 patients performed by The Cancer Genome Atlas (TCGA) consortium (TCGA Network 2015). This study established a framework for genomic classification of CM into four molecular subtypes based on the most frequently mutated driver gene: BRAF-mutated, NRS-mutated, NF1-loss, and triple-wild type.

### BRAF + melanomas

A mutation in the BRAF oncogene characterizes the BRAF melanoma subtype (B-Raf proto-oncogene). BRAF is a member of the *RAF* family of highly conserved serine/threonine kinases (A-Raf, B-Raf, and C-Raf) activated by Ras. They are components of the mitogen-activated protein kinase (MAPK) signalling pathway - the most frequent dysregulated pathway in human cancers, including malignant melanoma (Guo et al. 2020). Recurrent mutations in the *BRAF* gene were first identified in 2002 by Davies et al. (2002), who detected pathogenic variants in 59% of analyzed melanoma cell lines. Subsequent studies showed that *BRAF* mutation frequency ranges from 28 to 54% of analyzed melanoma samples depending on the analyzed group of patients, biological material, and technique (Ticha et al. 2019; Diefenbach et al. 2020). There are many pathogenic variants in the *BRAF* gene, which can be divided into three classes. Class I includes mutations within codon V600, which results in the constitutive activation of RAS-dependent monomers with high BRAF kinase activity. Class II comprises variants beyond codon 600, which cause constitutive activation of RAS-dependent dimers with high or moderate BRAF kinase activity. The last one is class III, which includes variants leading to low or no BRAF kinase activity (Dankner et al. 2018; Lokhandwala et al. 2019). The most common pathogenic variants of *BRAF* are V600E (62%) and V600K (14%). V600R/M/D/G mutations constitute approximately 5% of melanoma cases (Lokhandwala et al. 2019). These substitutions make the protein almost 500 times more active than the wild-type kinase, leading to constant activation of RAF/MEK signaling pathway (Gray-Schopfer et al. 2007). *BRAF* mutations are characterized by high variability even within a single patient. The status of this mutation can vary between primary tumor and metastasis (intertumour heterogeneity) as well as within a single tumor (intratumour heterogeneity) (Ito et al. 2021). It is estimated that approximately 30% of primary tumors have mutated *BRAF*, while the frequency of these mutations in metastatic melanoma is significantly higher, reaching 57% (Shinozaki et al. 2004). *BRAF* mutation is strongly associated with interval UV exposure and early outcomes; thus, it is more frequent in younger patients (Long et al. 2011; Newell et al. 2022). BRAF + subtype commonly manifests as superficial spreading melanoma on the trunk. They are more aggressive, have a higher predisposition to metastasis, and correlate with worse prognosis (Zablocka et al. 2022).

### RAS + melanomas

The RAS subtype of melanoma is characterized by mutations in the *RAS* gene family, including *NRAS*, *HRAS*, and *KRAS*. RAS proteins are membrane-bound small G proteins that, when activated, recruit RAF proteins to the membrane and exhibit various mechanisms of action through effectors such as members of the RAF family or PI3K. These proteins are part of MAPK and PI3K pathways, which regulate cell proliferation, differentiation, survival, migration, and apoptosis. Mutations in these kinases lead to the loss of GTPase enzymatic activity, resulting in the enzyme remaining in its active form (Malumbres and Barbacid 2003; Daud and Bastian 2012). *RAS* oncogenes are driver genes in about 30% of melanoma cases, with *NRAS* being the dominant one, accounting for 25–28% of melanoma cases (Hodis et al. 2012; TCGA Network et al. 2015; Lokhandwala et al. 2019; Louveau et al. 2020). The most common variant of *NRAS* mutation is in locus Q61, with Q61K and Q61R being the most frequent ones, accounting for 31% and 38% of cases, respectively (Lokhandwala et al. 2019). Since *BRAF* and *NRAS* proto-oncogenes activate the same pathway (MAPK), they are mutually exclusive. However, in approximately 2% of patients, they are detected concurrently (Siroy et al. 2015; Heppt et al. 2017). Interestingly, *RAS* mutations are more likely to coexist with classes 2 and 3 non-V600 *BRAF* mutations rather than with class 1 (Lokhandwala et al. 2019). Such a combination is detected in up to 18% of *BRAF* non-V600 melanomas (Siroy et al. 2015). It has been reported that in melanoma cells with mutated *RAS*, activation of the MAPK signalling pathway occurs preferentially through CRAF instead of BRAF (Dumaz et al. 2006; Bradish and Cheng 2014). This indicates CRAF as a potential therapeutic target in the RAS + melanoma subtype. *NRAS* mutation may also coexist with mutated *TP53* (17% of cases Siroy et al. 2015) and *PPP6C* (TCGA Network 2015).

### NF1 + melanomas

The third molecular subtype of melanoma is the NF1 + subtype, which is characterized by a deactivating mutation in the *NF1* gene. *NF1* encodes the neurofibromin 1 protein, which negatively controls RAS proteins. Loss of function (LOF) mutations in *NF1* lead to the activation of two key cancerogenesis pathways: RAS/MAPK and PI3K/AKT. NF1 + melanomas constitute about 12–23% of melanoma patients (TCGA Network 2015; Hayward et al. 2017; Newell et al. 2022) and are characterized by the highest mean rate of mutations (39 mutations/MB), stronger than BRAF + subtype UV signature correlation, and worst overall survival (TCGA Network et al. 2015; Cirenajwis et al. 2017). It also occurs more often in males than in females and more frequently in older people, with 72 years being the median age of diagnosis (Cirenajwis et al. 2017). The *NF1* mutation is more common in fast-growing melanomas with increased Breslow thickness and ulceration (de Unamuno Bustos et al. 2017). Despite the high mutation burden, identification of other actionable mutations in NF1 + melanomas is challenging (Rajkumar and Watson 2016). Potential candidates are the RASopathy genes, such as *RASA2*, *PTPN11*, or *RAF1* (Krauthammer et al. 2015; Cirenajwis et al. 2017). In addition, 70% of NF1 + subtypes in cutaneous melanoma have *TP53* aberrations (Newell et al. 2022), and approximately 30% have co-occurring mutations in *ARID2* (Rajkumar and Watson 2016). A higher frequency of *RB1* mutations has also been observed, according to TCGA Network (2015). Loss of *NF1* function co-occurres also with the mutated *BRAF* and *NRAS* melanomas. In the previous ones, this combination leads to resistance to RAF inhibitors (Nissan et al. 2014).

### Triple-negative melanomas

The fourth subtype is the triple wild-type melanoma. It is characterized by the absence of mutations in any of the aforementioned driver genes and occurs in about 5–8% of cases (TCGA Network et al. 2015). Unlike other subtypes, only 30% of triple wild-type melanomas show a correlation with the UV signature (Rajkumar and Watson 2016) and have the lowest TMB with only 9.2 mutations/Mb (TCGA Network et al. 2015; Rajkumar and Watson 2016; Hayward et al. 2017; Newell et al. 2022). However, triple wild-type melanoma has significantly more copy-number amplifications, complex structural rearrangements, and potential fusion drivers than the other subtypes (Rajkumar and Watson 2016). This subtype is also more likely to have a *KIT* mutation, which is present in about 20% of cases (TCGA Network et al. 2015; Newell et al. 2022). Other potential driver mutations include *PDGFRA*, *KDR*, *GNAQ*, and *GNA11*. This subtype also has a higher frequency of *CDK4* and *CCND1* mutations than the other melanoma molecular subtypes (TCGA Network et al. 2015).

## Genomic profiling of melanoma with NGS

Next-generation sequencing (NGS) has revolutionized cancer research and personalized cancer treatment. It offers several advantages over the traditional sequencing method: lower cost of sequencing than Sanger method, high throughput, and high sensitivity in detecting low-frequency variants (Sabour et al. 2017; Zhong et al. 2021).

In 2016, approximately ten years after the start of NGS profiling, Zhang and coauthors reported a summary of the results of whole genome and whole exome sequencing of melanoma (Zhang et al. 2016). Here, we would like to update the data. We have analyzed and compared the results of 12 studies aiming at genomic profiling of melanoma using various commercial (3) or custom (9), melanoma-specific (8), or universal (4) NGS panels. The criteria for selecting scientific articles were targeted sequencing of melanoma DNA samples, available information about the genomic panel, and variant allele frequency (VAF) in analyzed samples.

**Fig. 1:**
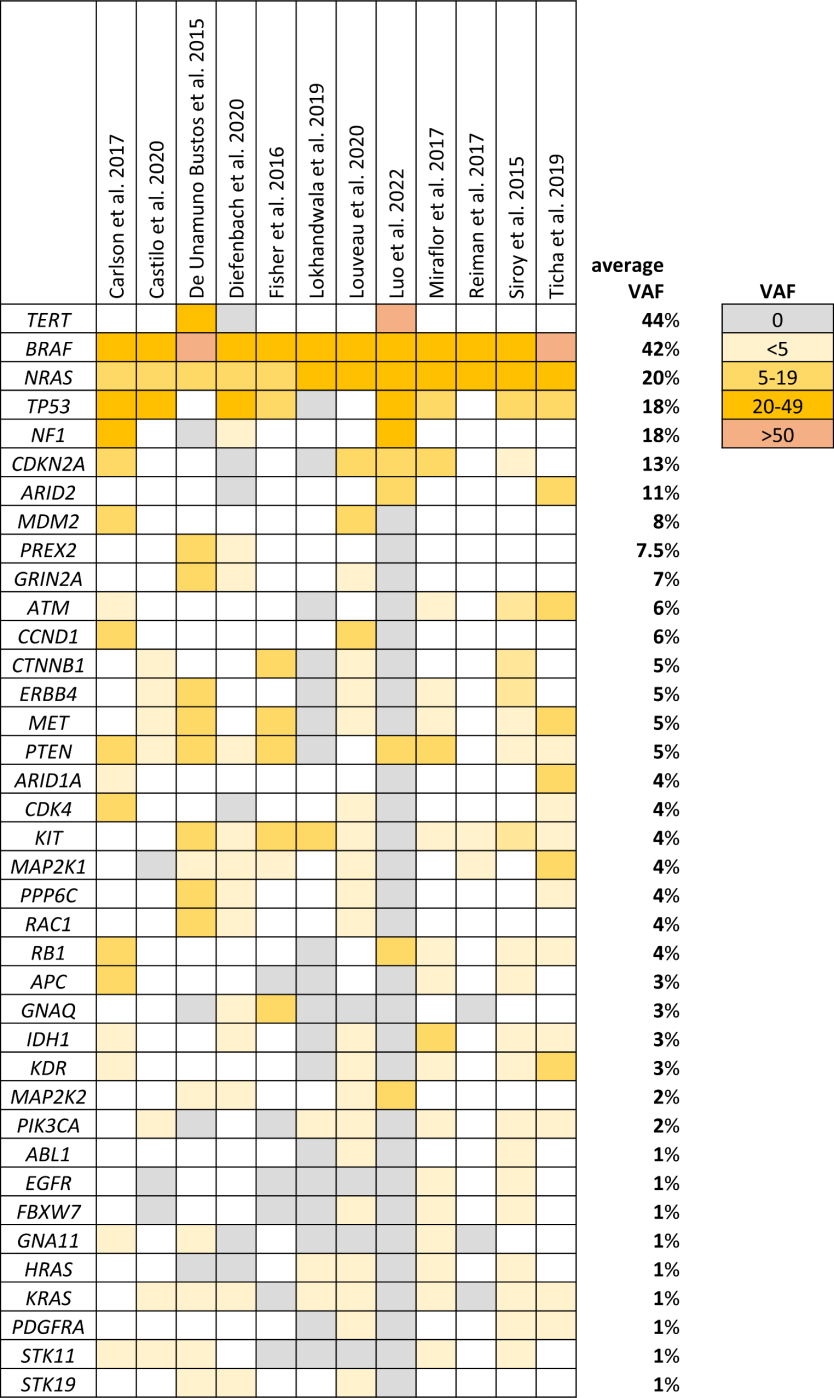
Most frequently mutated genes in melanoma in selected NGS genomic profiling studies. VAF – variant allele frequency; white colour denotes the lack of the gene in the panel

The number of genes in the panels ranged from 11 to 468 (mean: 90), and the number of patients (melanoma samples) from 25 to 911 (mean: 248). In all studies, the genetic material used for sequencing was DNA derived from formalin-fixed paraffin-embedded tumour samples (FFPE DNA). One study also used cell-free DNA (cfDNA) (Diefenbach et al. 2020). Figure 1 presents genes selected according to the following criteria: present in at least three panels, detected in at least two studies, and VAF > 1%. There are 38 genes, including those with high (> 10%, 7), medium (> 5%, 10), and low (< 5%, 21) frequency. Most genes have also been noted by Zhang et al. (2016) and have comparable mean VAF. However, some genes are significantly underrepresented in the panels used in the studies we analyzed (e.g. *NF1*, *PREX2*, *PTPRK*, *DCC*). *DCC* and *PTPRK* were detected with relatively high frequency in WES studies (mean 21.5% and 11.5%, respectively), but they were omitted entirely in the targeted panels. On the other hand, in our study set, there are genes not analyzed in the summary Table 1 by Zhang et al. (2016), e.g. *CTNNB1*, *CCND1*, *ATM*, *MET*, *APC*, and *KDR*. The most underrepresented genes in the panels are *NF1* and *TERT* (promoter). The *NF1* gene was present in four panels and detected in three studies. *NF1* comprises 58 exons and has no mutation hotspots (Pasmant et al. 2015). The necessity of sequencing whole gene/coding sequences could be the reason for not including this gene in the panel. The shortcoming of the analyzed studies is also the lack of copy number variation (CNV) analysis, which was performed only in one study (Louveau et al. 2020). Therefore, deleted or amplified genes may be underrepresented e.g. *PTEN*, *CDKN2A*, *MITF*, and *CCND1*, compared to the results obtained e.g. by Hodis et al. (2012). A different situation occurs with the *TERT* promoter. It was present in three panels and detected in two studies. Technical problems with the amplification of this DNA region due to high guanine-cytosine (GC) content are to blame for its absence in the studies (Lee et al. 2022). The difference between the results of Zhang et al. comparison analysis and ours concerns the VAF of analyzed genes in samples. The following ones have lower mean VAF in targeted profiling studies than in Zhang et al. summary: *PPP6C*, *GRIN2A*, *ARID2*, *APC*, *GNAQ*, *EGFR*, *FBXW7*. Despite some shortcomings of the analyzed studies, we have selected 22 genes to be included in the melanoma genomic panel. Six of them, namely *PREX2*,* IDH1*,* ERBB4*,* PIK3CA*,* FBXW7*, and *PPP6C* are described more thoroughly as they are relatively new, NGS-derived genes, and the available functional data suggests their potential clinical significance.

**Table 1:** Our melanoma gene panel proposal

Gene symbol	Mutation	Biological significance	Clinical significance
*ARID2*	LOF mutations	Cell cycle control, tumour suppressor	Predictive marker, response to immunotherapy
*BRAF*	Hotspot mutations: V600E, V600K, V600D	Proliferation, oncogene	Melanoma driver gene, molecular subtype identification, therapeutic target
*CCND1*	Amplification	Cell cycle control, oncogene	Predictive marker, resistance to BRAFi/MEKi
*CDK4*	Amplification	Cell cycle control, oncogene	Therapeutic target, resistance to BRAFi/MEKi
*CDKN2A*	Deletion	Cell cycle control, tumour suppressor	Resistance to BRAFi/MEKi, poorer overall survival, potential targeted therapy
*CTNNB1*	GOF mutations	Immune escape, oncogene	Predictive markers, response to immunotherapy
*EGFR*	Amplification	Proliferation, oncogene	Poor survival prognosis, therapeutic target
*ERBB4*	GOF mutations, “mini hotspot”: E452K	Proliferation, oncogene	Potential therapeutic target
*FBXW7*	LOF mutations, most frequent in codon: R465	Migration, angiogenesis, tumour suppressor	Predictive markers, response to immunotherapy
*IDH1*	Hotspot mutation: R132	Proliferation, oncogene	Potential therapeutic target
*KIT*	Amplification / L576P, K642E	Proliferation, oncogene	Melanoma driver gene, therapeutic target
*MAP2K1*	GOF mutations: P124S, E203K	Proliferation, oncogene	Predictive marker, resistance to BRAFi/MEKi
*MDM2*	Amplification	Cell cycle control, oncogene	Potential therapeutic target
*MET*	Amplification	Survival, migration, oncogene	Therapeutic target, resistance to BRAFi/MEKi
*NF1*	LOF mutation/deletion	Anti-apoptosis, tumour suppressor	Melanoma driver gene, molecular subtype identification
*NRAS*	Hotspot mutations: Q61R, Q61K, Q61L, Q61H	Proliferation, oncogene	Melanoma driver gene, molecular subtype identification, potential therapeutic target
*PIK3CA*	Hotspot mutations: H1047R, E545K	Proliferation, survival, oncogene	Resistance to BRAFi /MAPKi, potential therapeutic target
*PPP6C*	Hotspot mutation: R264C	Cell cycle control, tumour suppressor gene	Predictive marker, resistance to BRAFi/MEKi
*PREX2*	Amplification	Oncogene	Potential therapeutic target
*PTEN*	LOF mutations/deletions	Tumor suppressor	Predictive marker, resistance to BRAFi/MEKi, response to immunotherapy
*RAC1*	Hotspot mutation: P29S	Proliferation, oncogene	Predictive marker, resistance to BRAFi/MEKi
*TP53*	LOF mutations	Apoptosis, tumour suppressor	Melanoma driver gene

### PREX2

PREX2 is a phosphatidylinositol (3,4,5)-trisphosphate-dependent Rac exchanger 2 (P-Rex2) –a small (183 kDa) protein that activates GTPase - RAC1. It is present in all cell types, except neutrophils, and regulates Ras signaling (Donald et al. 2004). PREX2 binds tumor suppressor PTEN, leading to activation of the PI3K/AKT pathway (Fine et al. 2009). It was initially found to be mutated in 14% of melanomas (Berger et al. 2012). Higher frequency was noted by Zhang et al. (2016), while lower frequency was noted in our summary (Fig. 1). Truncating mutations in this gene activate its oncogenic potential and lead to increased proliferation of NRAS-mutated melanoma cells by activating the PI3K/AKT pathway and RAC1 (Deribe 2016). PREX2 was also proven to induce the invasion of breast cancer cells and mouse embryonic fibroblasts (Mense et al. 2015). Besides melanoma, *PREX2* is also a potential driver of pancreatic (Waddell et al. 2015) and hepatocellular cancer (Yang et al. 2019). Oncogenic activity and the relatively high frequency of its mutated form in melanoma make PREX2 a potential therapeutic target and a candidate for melanoma-diagnostic panel.

### ERBB4

ERBB4 (HER4) is a human epidermal growth factor receptor 4 and belongs to the ERBB protein family (ERBB1-ERBB4), which is a type I tyrosine kinase subfamily. They regulate the growth, survival, differentiation, and apoptosis of the cells. *ERBB4* is expressed in many human tissues and is mutated in such cancers as colorectal (CRC), non-small cell lung (NSCLC), or breast cancer (Segers et al. 2020). The mutation rate of *ERBB4* in melanoma is 19% and there is one ”mini-hotspot” (E452K) (Prickett et al. 2009). *ERBB4* mutations are of gain-of-function (GOF) type and lead to increased autophosphorylation of the receptor and protein tyrosine kinases (PTKs) activity (Prickett et al. 2009). The proliferation of melanoma cells harboring mutant *ERBB4* is driven by the ERBB4 signaling pathway, and inhibition of this protein causes melanoma cell death (Rudloff and Samuels 2010). Thus, mutated ERBB4 is a potential therapeutic target in melanoma treatment (Prickett et al. 2009; Mancini et al. 2019).

### PPP6C

*PPP6C* (protein phosphatase 6 catalytic subunit) encodes the catalytic subunit of the PP6 serine-threonine phosphatase complex. It is a member of the PPP family, which is involved in DNA damage repair and cell cycle regulation (Ohama 2019). The frequency of mutations in this gene in melanoma, according to TCGA Network (2015), is 6–9%, and according to Fig. 1 is not more than 5%. Loss of *PPP6C* destabilizes the PP6 complex, leading to chromatin instability and DNA damage (Hammond et al. 2013). It also causes MEK hyperphosphorylation, which results in sustained ERK activity and resistance to MEKi (Cho et al. 2021). This data suggests that *PPP6C* is a melanoma suppressor gene (Hammond et al. 2013). On the other hand, the most common variant, R264C, is a gain-of-function mutation found in 22% of PPP6-mutated metastatic melanomas (Maskin et al. 2022). In the zebrafish model, this variant, in cooperation with oncogenic *NRAS* (Q61K) was proved to modulate MITF expression, leading to increased melanocyte proliferation, survival, invasiveness, and resistance to BRAFi (Maskin et al. 2022). This data, in turn, suggests its oncogenic role. *PPP6C* pathogenic variants co-occur predominantly with *BRAF* and *NRAS* mutations with ∼ 55% (Krauthammer et al. 2012, 2015) and 18% frequency (Xia et al. 2014), respectively. Interestingly, male patients with mutated *PPP6C* have a better survival outcome than females with the same variant (Shi et al. 2021).

### FBXW7

*FBXW7* (F-box and WD repeat domain-containing 7) encodes a member of the F-box protein family, a component of the SCF complexes (Skp1–Cul1–F-box protein–Rbx1). It recognizes and binds such proteins as c-Myc, cyclin E, or c-Jun, which play an essential role in cell growth, proliferation, differentiation, and survival. FBXW7 is a known tumour suppressor, and its loss leads to cancer development due to the disruption of oncoproteins degradation and chromosome instability (Akhoondi et al. 2007; Cheng and Li 2012). Mutations in *FBXW7* commonly occur in colorectal (CRC), esophageal, and gastric cancers (ESCC) (Yeh et al. 2018). In melanoma patients, mutated *FBXW7* was first detected by Aydin et al. (2014) with a frequency of 8%. According to Fig. 1, the frequency of mutations in this gene is approximately 4–6%. The expression level of *FBXW7* decreases with the stage of melanoma and depends on the morphological type of tumour and tumour invasion. It is significantly lower in advanced melanoma, stage pT3/pT4, than in dysplastic nevi or melanoma in situ and stage pT1/pT2 (Mozuraitiene et al. 2020). A low level of *FBXW7* expression is associated with increased melanoma cell migration, metastatic potential (Cheng et al. 2013) and increased levels of genes promoting tumour angiogenesis (Aydin et al. 2014). FBXW7 may also be involved in resistance to immune checkpoint inhibitors. Gstalder et al. (2020) observed in a mouse model that tumours resistant to immunotherapy had a LOF mutation in *FBXW7*, in contrast to tumours that responded to the treatment.

### PIK3CA

*PIK3CA* (phosphatidylinositol-4,5-bisphosphate 3-kinase catalytic subunit alpha) encodes the p100α protein (124 kDa), which is a component of the PI3K-AKT signaling pathway. Mutations in *PIK3CA* are commonly observed in breast and colorectal cancers, with frequency of 36% and 18%, respectively (The AACR Project GENIE Consortium et al. 2017). In melanoma, *PIK3CA* mutations are rare and occur in 2–3% of patients. There are two primary *PIK3CA* mutations, H1047R and E545K, which lead to the activation of PI3K/AKT pathway (Omholt et al. 2006). H1047R mutation not only sustains proliferation and cell survival but also confers resistance to targeted melanoma therapy, which was shown in in vitro and murine models (Deuker et al. 2015; Silva et al. 2017; Candido et al. 2022). In turn, the *PIK3CA* E545K mutation pre-existing in NRAS-driven melanoma was indicated as an aberration that caused resistance to MEK and CDK4/6 inhibitors in melanoma patients (Romano et al. 2018). Therefore, inhibition of mutated *PIK3CA* in combination with BRAF/MEK inhibitors may extend the response to this treatment. Several inhibitors targeting the PI3K pathway have been approved by the FDA (copanlisib, idelalisib, umbraniesib, duvelisib, alpelisib), and many other compounds are being tested in various cancers, including melanoma (Mishra et al. 2021).

### IDH1

*IDH1* encodes the isocitrate dehydrogenase 1 (NADP+) protein (46,7 kDa). IDH1 catalyzes the oxidative decarboxylation of isocitrate to α-ketoglutarate, which maintains DNA and histone proteins in a demethylated state. The heterozygous oncogenic gain-of-function mutations in *IDH1* cause the inhibition of histone demethylation, leading to the inhibition of differentiation, which promotes tumorigenesis (Bruce-Brand and Govender 2020). Mutations in *IDH1* frequently occur in gliomas (60–80%), AML (20%), and chondrosarcoma (38–86%) (Tian et al. 2022). In melanoma patients, mutant *IDH1* occurs with a frequency of 3-5% (Fig. 1), with the most common hotspot in codon R132 (Linos and Tafe 2018). Significant co-occurrence of *IDH1* mutations with mutated *NRAS* was observed (Linos and Tafe 2018; Ticha et al. 2019). Mutated *IDH1* is more frequently detected in metastatic samples, suggesting a contribution to metastasis by e.g. enhancing the activation of the MAPK and STAT3 signaling pathways (Shibata et al. 2011). IDH1 inhibitors are currently used in treating AML, gliomas, and solid tumours. IDH1-positive melanoma patients are also potential beneficiaries of this treatment, significantly since inhibition of wild-type IDH1 improved melanoma response to chemotherapy (Zarei et al. 2022).

## Our melanoma gene panel

Based on the melanoma genomic profiling data presented in Fig. 1 we propose a 22-gene melanoma panel with potential clinical application (Table 1). The selected genes were divided into three groups: known melanoma driver genes, candidates for therapeutic targets, and candidates for predictive markers. Some genes fall into more than one set. The first group comprises *BRAF*, *NRAS*, *NF1*, *TP53*, and *KIT*. According to Fig. 1 all of them, except *KIT*, are mutated in more than 10% of melanoma cases. Analysis of the *BRAF*, *NRAS*, and *NF1* genes in patient samples will allow for the classification of melanoma into four proposed molecular subtypes (TCGA Network et al. 2015). Although so far, only *BRAF* is targetable, the establishment of *NRAS* and *NF1* status is important as it will allow further research on the influence of these genes on the clinical behavior of melanoma. Furthermore, many studies are testing various experimental treatments for NRAS + melanoma patents (Randic et al. 2021). In contrast to *NRAS*, *KIT* is a well-established therapeutic target with many approved inhibitors (e.g. imatinib, ripretinib). It is also tested in melanoma (Guo et al. 2017). Although this oncogene is activated only in a few percent of melanoma cases, for the sake of KIT + melanoma patients, it should be a part of the melanoma panel. The last gene in this group is *TP53*. It is mutated in a relatively low number of melanomas compared to other cancers. However, its function is suppressed in most cases of melanoma. One of the mechanisms is its increased inactivation and degradation caused by the *MDM2* (mouse double minute 2) or *CDKN2A* mutations. MDM2 is the main ubiquitin ligase involved in the degradation and inactivation of p53. Genetic aberrations of *MDM2* (mainly amplifications) leading to increased expression of this protein are present in approximately 3% of melanoma cases (according to Fig. 1–8%). They are more prevalent in advanced melanoma and associated with worse prognosis (Khan et al. 2023). Therefore, *MDM2* as an oncogene is a potential therapeutic target whose inhibition can restore p53 activity. The p14ARF protein encoded by *CDKN2A* directly inhibits MDM2. If *CDKN2A* is deleted, which occurs in more than 10% of melanomas, MDM2 strongly inhibits p53 leading to tumour progression. Therefore, deletions of the *CDKN2A* gene could also qualify for targeted therapy with MDM2 inhibitors or other p53-reactivating agents with antitumor activity (Loureiro et al. 2021). Those two aforementioned genes fall into the second group of genes in the panel: candidates for targeted therapy. Other actionable mutations occur in *PIK3CA*, *IDH1*, *ERBB4*, *PREX2*, *EGFR*, *CDK4*, *MET*, and *PPP6C*. Mutated *ERBB4*, *EGFR*, *CDK4*, *PIK3CA*, *IDH1*, and *MET* are well-established therapeutic targets in various cancers, including breast (Hunter et al. 2023), *CDK4*), lung (Zubair and Bandyopadhyay 2023), *EGFR*), (Mathieu et al. 2022), *MET*), lymphomas (Yu et al. 2023), *PIK3CA*), and leukemia (Chen et al. 2023), *IDH1*), however, their inhibitors are not yet recognized in melanoma treatment. Some of them have been tested in melanoma, e.g., *CDK4* (Guo et al. 2020), but without clinical success so far. Apparently, the low frequency of these pathogenic variants in melanoma is to blame for the low interest in their therapeutic targeting in this malignancy. However, if mutation-directed therapy is to be implemented in the clinic, these genes, in our opinion, should be a part of the melanoma gene panel. We also propose to include two completely new genes as potential therapeutic targets in the panel: *PREX2* and *PPP6C*, which were described above.

The third group comprises genes whose mutational profile may help predict the response to targeted therapy with BRAF/MEK inhibitors and immunotherapy with checkpoint immune control inhibitors. They are *PPP6C*, *CDKN2A*, *CCND1*, *MAP2K1*, *RAC1*, and *PIK3CA* (targeted therapy resistance), *ARID2* and *CTNNB1* (immunotherapy resistance), *PTEN* (both therapies). Some variants in *MAP2K1* and *RAC1* (see Table 1) are relatively well-described as causing resistance to BRAF/MEK inhibitors (e.g. Nikolaev et al. 2012; Watson et al. 2014; respectively) and are assigned as pathogenic (*MAP2K1*) or likely pathogenic (*RAC1*) in ClinVar database. Both aforementioned genes, together with *CDKN2A* and *PTEN* have been thoroughly described in our previous review paper (Olbryt 2022) as potential biomarkers of the response to the targeted therapy, while the biological and potential clinical significance of *PPP6C* and *PIK3CA* were presented above. Although the response to immunotherapy appears modulated by many factors (Olbryt et al. 2020), the genetic background of the tumor may also matter. Our candidate genomic determinants of the melanoma response to immunotherapy are *PTEN*, *ARID2*, and *CTNNB1*. We already described them in another review paper (Olbryt et al. 2020), and here we would only like to update the data. The association of *PTEN* loss with resistance to immunotherapy was generally confirmed in various recent studies using short-term tumor cell lines and matched tumor samples from melanoma patients progressing on immune checkpoint inhibitors (Lim et al. 2023), melanoma patients’ material (Cabrita et al. 2020) and also preclinical models of prostate cancer (Lin et al. 2021). It appears that loss of *PTEN* may be a potential marker of response to immunotherapy; however, apparently, together with other genomic/molecular factors rather than as a single biomarker. The role of *ARID2* is less prominent. It was first identified as a novel melanoma gene in 2012 mutated in 9% of samples (Hodis et al. 2012). Its LOF aberration in melanoma was further confirmed in other genomic studies with similar frequency (Zhang et al. 2016) and suggested tumor suppressor function. On the other hand, its decreased expression sensitizes melanoma cells to killing by T cells (Pan et al. 2018). Furthermore, *ARID2*-knockout melanoma tumors were proved to be more sensitive to immunotherapy in a murine model (Fukumoto et al. 2021). Finally, association analysis performed on more than 1500 melanoma samples showed that *ARID2* mutation correlates with higher immune response markers such as TMB (tumor mutational burden), PDl-1 expression, and dMMR/MSI-H compared to *ARID2*-WT (Akinjiyan et al. 2024). The aforementioned data would suggest an immune-suppressive function of ARID2A and the potential predictive significance of *AIRD2* LOF. However, although *ARID2* deficiency correlated with more prolonged overall survival, there was no difference in OS in patients treated with PD-1 inhibitors (Akinjiyan et al. 2024). Similar results were obtained for the correlation of mutated *APC/CTNNB1* with prognosis. Survival analysis of patients from TCGA-SKCM database revealed a worse prognosis for patients with *APC/CTNNB1* mutations compared to wild-type patients. However, there was no difference in response to immunotherapy between those groups (Karachaliou et al. 2022). Activating mutations in *CTNNB1*, especially in exon 3, were detected in many cancers, including melanoma (Kim and Jeong 2019). Previous results suggested that activation of the WNT/β-catenin pathway is involved in immunosuppression, as was described in our review (Olbryt et al. 2020), and mutated *CTNNB1* causes apoptotic resistance (Braggio et al. 2020). Furthermore, mutated *CTNNB1* is also a potential marker of response to targeted therapy (imatinib) (Cho et al. 2017). The lack of prognostic and predictive significance of *CTNNB1* and *ARID2* as individual predictors does not eliminate them as potential biomarkers. The complexity of immune response requires multi-factorial prognostic and predictive tests. Therefore, the genetic immune-response panel would involve apart from *CTNNB1* and *ARID2* also, *PTEN* (see above), and maybe *EGFR*, and *TP53* (Hilke et al. 2020). The last two are already in our panel as therapeutic targets.

The proposed panel takes advantage of the most up-to-date knowledge on melanoma genomics. It is intended to successfully companion the diagnosis, prognosis, monitoring, and selection of the best therapy tailored to the genomic profile of melanoma.

## Data Availability

No datasets were generated or analysed during the current study.
