# Correction: Melanoma genomics– will we go beyond *BRAF* in clinics?

**DOI:** 10.1007/s00432-025-06112-1

**Published:** 2025-02-13

**Authors:** Justyna Mirek, Wiesław Bal, Magdalena Olbryt

**Affiliations:** 1 Center for Translational Research and Molecular Biology of Cancer, Maria Sklodowska-Curie National Research Institute of Oncology Gliwice Branch, Gliwice, 44-101 Poland; 2 Chemotherapy Day Unit, Maria Sklodowska-Curie National Research Institute of Oncology Gliwice Branch, Gliwice, 44-101 Poland

**Correction: Journal of Cancer Research and Clinical Oncology (2024) 150:433**

10.1007/s00432-024-05957-2

In the BRAF + melanomas section, the article originally read ‘Class I includes mutations within codon V600, which results in the constitutive activation of RAS-dependent monomers with high BRAF kinase activity. Class II comprises variants beyond codon 600, which cause constitutive activation of RAS-dependent dimers with high or moderate BRAF kinase activity. The last one is class III, which includes variants leading to low or no BRAF kinase activity (Dankner et al. 2018; Lokhandwala et al. 2019)’. The article should instead have read ‘Class I includes mutations within codon V600, which results in the constitutive activation of RAS-independent monomers with high BRAF kinase activity. Class II comprises variants beyond codon 600, which cause constitutive activation of RAS-independent dimers with high or moderate BRAF kinase activity. The last one is class III, which includes variants leading to low or no BRAF kinase activity (Dankner et al. 2018; Lokhandwala et al. 2019)’.

The original article has been corrected.

